# *In vivo* Direct Conversion of Astrocytes to Neurons Maybe a Potential Alternative Strategy for Neurodegenerative Diseases

**DOI:** 10.3389/fnagi.2021.689276

**Published:** 2021-08-02

**Authors:** Youcui Wang, Xiaoqin Zhang, Fenghua Chen, Ning Song, Junxia Xie

**Affiliations:** Institute of Brain Science and Disease, School of Basic Medicine, Shandong Provincial Collaborative Innovation Center for Neurodegenerative Disorders, Shandong Provincial Key Laboratory of Pathogenesis and Prevention of Neurological Disorders, Qingdao University, Qingdao, China

**Keywords:** astrocytes, neurons, neurodegenerative diseases, *in vivo*, conversion

## Abstract

Partly because of extensions in lifespan, the incidence of neurodegenerative diseases is increasing, while there is no effective approach to slow or prevent neuronal degeneration. As we all know, neurons cannot self-regenerate and may not be replaced once being damaged or degenerated in human brain. Astrocytes are widely distributed in the central nervous system (CNS) and proliferate once CNS injury or neurodegeneration occur. Actually, direct reprogramming astrocytes into functional neurons has been attracting more and more attention in recent years. Human astrocytes can be successfully converted into neurons *in vitro*. Notably, *in vivo* direct reprogramming of astrocytes into functional neurons were achieved in the adult mouse and non-human primate brains. In this review, we briefly summarized *in vivo* direct reprogramming of astrocytes into functional neurons as regenerative strategies for CNS diseases, mainly focusing on neurodegenerative diseases such as Parkinson’s disease (PD), Alzheimer’s disease (AD), and Huntington’s disease (HD). We highlight and outline the advantages and challenges of direct neuronal reprogramming from astrocytes *in vivo* for future neuroregenerative medicine.

## Introduction

Partly because of extensions in lifespan, the incidence of neurodegenerative diseases such as Alzheimer’s disease (AD), (Alzheimer’s disease facts and figures, Alzheimers Dement, 2021), Parkinson’s disease (PD), Huntington’s disease (HD), amyotrophic lateral sclerosis (ALS), is increasing, and these neurodegenerative diseases result in a heavy social and economic burden in the world. However, the pathological mechanisms underlying neurodegenerative diseases are unclear. A variety of factors such as genetic, environmental, and aging factors, are considered to participate in the neurodegeneration (Przedborski, 2017). Abnormal protein aggregation (Spires-Jones and Hyman, 2014; Wang et al., 2020), oxidative stress with reactive oxygen species (ROS) (Xu et al., 2016; Bi et al., 2018; Singh et al., 2019; Wang et al., 2020), iron deposition (Jiang et al., 2017; Levi et al., 2019; Chen et al., 2021b), mitochondrial dysfunction (Przedborski, 2017; Wang et al., 2020), autophagy (Chen et al., 2017, 2021a; Ren et al., 2021), decreasing of neurotrophins (Allen et al., 2013), and neuroinflammatory responses (Kwon and Koh, 2020), are recognized as common pathophysiological mechanisms, and all of those contribute to the neuronal loss in brains. Thus far, there are no effective strategies to slow neuronal degeneration, or prevent the progression of them. Logically, replenishing the lost neurons can repair the neuronal function by endogenous neurogenesis or cell transplantation (Chen et al., 2019). As we all know, neurons cannot self-regenerate, while glial cells such as astrocytes can proliferate upon injury or disease (Li and Chen, 2016; Chen et al., 2019; Lei et al., 2019). Currently, neurons may not be replaced once being damaged or degenerated in human brain (Li and Chen, 2016; Boldrini et al., 2018; Lei et al., 2019), and there is a debate on whether adult human brains have endogenous neurogenesis (Lei et al., 2019). The newborn neurons are largely restricted in a few regions such as the hippocampus and the subventricular zone (Boldrini et al., 2018; Sorrells et al., 2018), and the progenitor cells migrating toward injury sites often differentiate into glia rather neurons (Faiz et al., 2015), which has disadvantageous effect on repairing damaged or degenerative brains. Nevertheless, numerous studies have shown that embryonic stem cells (ESCs) or induced pluripotent stem cells (iPSCs)-derived transplants can survive, innervate, produce neurotransmitters, and promote functional recovery in animal models (De Gioia et al., 2020; Gantner et al., 2020; Song et al., 2020), and even clinical trials with those transplants have achieved some success (Kefalopoulou et al., 2014; Schweitzer et al., 2020). There are several serious problems that are difficult to overcome including supply and availability, ethical issues, immune rejection for ESCs, and the risk of tumor formation, genomic stability for iPSCs (Trounson and McDonald, 2015; Martin, 2017).

Shinya Yamanaka’s team was pioneered in the ground-breaking iPSC technology that mouse fibroblasts could be reprogrammed into the pluripotent stem cells by delivering four transcription factors (TFs) (Oct4, Sox2, Klf4, and c-Myc) *in vitro* (Takahashi and Yamanaka, 2006). Then, it was reported that human somatic cells can be converted into human iPSCs (hiPSCs) in the following years (Takahashi et al., 2007; Park et al., 2008). HiPSCs technology has no ethical concern and immune rejection, showing great promise in the regenerative strategies by mass-generating patient-specific stem cells (Stadtfeld and Hochedlinger, 2010). Based on the hiPSCs technology (Stadtfeld and Hochedlinger, 2010), direct lineage reprogramming, which converts a specific somatic cell type to another, is developed rapidly. The cells from direct lineage reprogramming are not passing through a pluripotent state, therefore, they mostly have no the ability of tumor formation (Chambers and Studer, 2011). Thereafter, it has been reported that various types of functional cells, including neurons, that can be successfully generated by a direct reprogramming strategy *in vitro* and even *in vivo* (Vierbuchen et al., 2010; Niu et al., 2013), which leads to the understanding of direct reprogramming technology as an alternative approach for rescuing neurodegeneration.

The primary consideration is the starting cell type in generating neurons via direct lineage reprogramming. Given that fibroblasts have large quantity and extensive distribution in the body, they are promising candidate cells for reprogramming, and indeed by introducing the combined expression of neural lineage-specific TFs, direct reprogramming of mice and human fibroblasts into neurons was first reported *in vitro* (Vierbuchen et al., 2010). However, considering the fact that (1) glial cells are proximal in lineage distance to neurons and ubiquitous distribution in adult mammalian brains; (2) they constitute over half of the total brain cells; (3) they have intrinsic proliferative capability, glial cells can serve as ideal source cells for reprogramming into neurons to achieve neuroregeneration (Amamoto and Arlotta, 2014; Guo et al., 2014). Astrocytes serve with multiple important physiological functions in the central nervous system (CNS), such as the constitution and maintenance of the blood-brain barrier (Figley and Stroman, 2011), producing and releasing of neurotrophic factors (Anderson et al., 2016), adjusting the density of ion in the extracellular space (Walz, 2000), modulating neuroinflammation (Kwon and Koh, 2020; Vadodaria et al., 2021), and even regulating neuronal activity, synaptic transmission, and neural circuit function (Bazargani and Attwell, 2016; Li et al., 2020; Vadodaria et al., 2021; Yin et al., 2021). Although astrocyte proliferation largely stops after 1 month of age in rodents (Ge et al., 2012), in response to insults, including brain trauma, stroke and neurodegeneration, astrocytes often are under a state that called astrogliosis, in which astrocytes accelerate proliferation (Liddelow and Barres, 2017). For the first time, the approach for reprogramming astrocytes into neurons has been found by high expression of the neurogenic TF Pax6 *in vitro* (Heins et al., 2002). Indeed, astrocytes originating from the same progenitor cells with neurons (Kriegstein and Alvarez-Buylla, 2009) are efficiently reprogrammed into functional neurons with one TF (Heins et al., 2002; Berninger et al., 2007). The above studies indicate that astrocytes may be an ideal starter cells for reprogramming into neurons to replace the lost neurons in neurodegenerative diseases.

It is fascinating that these reactive astrocytes are *in situ* reprogrammed into functional neurons for neural circuit reestablishment and functional recovery. Actually, direct reprogramming astrocytes into functional neurons has been attracting more and more attention in recent years (Amamoto and Arlotta, 2014; Chen et al., 2015). It has been confirmed that human astrocytes can be successfully converted into neurons or neuroblasts *in vitro* (Corti et al., 2012; Zhang et al., 2015; Li et al., 2016; Rivetti di Val Cervo et al., 2017; Yin et al., 2019; Qian et al., 2020). These studies provide a potential alternative approach to regenerate functional new neurons in CNS of adult mammalian by *in situ* directly reprogramming astrocytes into neurons. Notably, *in vivo* direct reprogramming of astrocytes into functional neurons were achieved in the adult mouse brain, and even in the adult non-human primate brains (Guo et al., 2014; Chen et al., 2015, 2020; Liu et al., 2015; Niu et al., 2015; Li and Chen, 2016; Srivastava and DeWitt, 2016; Brulet et al., 2017; Rivetti di Val Cervo et al., 2017; Qian et al., 2020; Wu et al., 2020; Zhou et al., 2020). In this review, we briefly summarized *in vivo* direct reprogramming of astrocytes into functional neurons as regenerative strategies for CNS diseases, mainly focusing on neurodegenerative diseases such as PD, AD, HD, and ALS. We highlight and outline the advantages and challenges of direct neuronal reprogramming *in vivo* for future neuroregenerative strategies.

## *In vivo* Astrocyte-to-Neuron Conversion for PD

Parkinson’s disease (PD) is the second common progressive neurodegenerative after AD, and its main pathological characteristics are the progressive loss of dopaminergic neurons in the substantia nigra region and the depletion of dopamine in the striatum, which cause some major clinical motor symptoms of PD including rest tremor, rigidity, bradykinesia, and postural instability (Przedborski, 2017). So far, there is no effective treatment strategy that can protect dopaminergic neurons from neurodegeneration.

Evidence has been provided that the transplantation of dopaminergic neurons derived from the human fetal ventral midbrain, were able to partially improve the clinical motor function in PD patients (Kefalopoulou et al., 2014; Li and Chen, 2016). However, the clinical transplantation of those dopaminergic neurons is limited because of the ethical issues in harvesting human fetal tissue, immune rejection, and the risk of transplantation-induced dyskinesia (Olanow et al., 2003). In order to obtain standardizing dopaminergic neurons for clinical application, an alternative approach is to induce iPSCs into dopaminergic neurons *in vitro*. Those dopaminergic neurons have no immune rejection for transplantation, but have the risk of tumor if not properly controlled (Kwon and Koh, 2020). These induced dopaminergic neurons are capable to restore certain function after transplantation in animal models of PD (Chen et al., 2019). Furthermore, the transplantation of dopaminergic neurons derived from iPSCs has been achieved in a clinical trial (Schweitzer et al., 2020).

Initially, Caiazzo et al. (2011) reported that mouse and human fibroblasts can be directly converted into functional dopaminergic neurons called ‘induced dopamine-releasing’ (iDA) neurons *in vitro* by forced expression of three transcription factors: Mash1 (also called Ascl1), Lmx1a and Nurr1 (also called Nr4a2), together referred as ALN, in which lentiviruses expressing three factors were employed. Ascl1, Lmx1a, and Nurr1 are critical for midbrain dopaminergic neurons development (Arenas et al., 2015), and the reprogramming of astrocytes into dopaminergic neurons has been successful *in vitro* by delivering these three transcription factors in a single polycistronic lentiviral vector (Addis et al., 2011). However, the ALN combination converted astrocytes or NG2 glia into GABAergic neurons, rather into dopaminergic neurons in mouse brains by using the Cre-recombinase-dependent AAV vectors (Torper et al., 2015). Afterward, Rivetti di Val Cervo et al. (2017) reported a strategy that was able to generate induced dopaminergic neurons from human astrocytes with reprogramming efficiency reaching up to 16% *in vitro* by using overexpression of three transcription factors in a lentiviral vector, NeuroD1, Ascl1, and Lmx1a, and the microRNA miR-218, collectively called NeAL218. Moreover, in a mouse model of PD, adult striatal astrocytes transfected by lentiviruses with overexpression of the NeAL218 factors were also converted into dopaminergic neurons, and those induced dopaminergic neurons promoted the motor improvement (Rivetti di Val Cervo et al., 2017; Fyfe, 2017).

**FIGURE 1:**
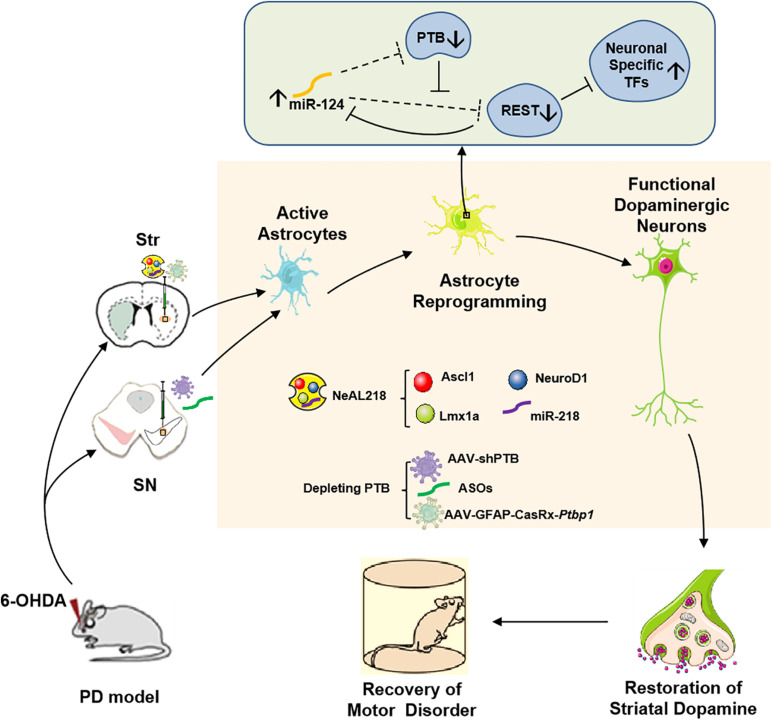
*In vivo* reprogramming of astrocytes into functional dopaminergic neurons promotes behavioral improvement in a mouse model of PD. Astrocytes can be converted into dopaminergic neurons by high expressing three transcription factors (NeuroD1, Ascl1, and Lmx1a), and miR-218, collectively referred to as NeAL218 in mouse striatum (Fyfe, 2017; Rivetti di Val Cervo et al., 2017) or by depleting an RNA-binding protein called PTB in mouse striatum or substantia nigra (Qian et al., 2020; Zhou et al., 2020), which leads to behavioral improvement in a mouse model of PD. The molecular mechanism of astrocyte-to- dopaminergic neuron conversion by depletion PTB is that RE1-silencing transcriptional factor (REST) complex prevents the expression of multiple neuron-specific transcriptional genes (for example Ascl1, NeuroD1) as well as miR-124 in astrocytes (Hu et al., 2018). Although REST also is the target of miR-124 in such loop, such targeting is potently inhibited by PTB via direct competition of miR-124 targeting on REST components. Thus, depletion of PTB can promote miR-124 to efficiently target REST (Qian et al., 2020). Considering that miR-124 also is able to target PTB, the derepression of miR-124 can further inhibits PTB and REST, leading to the expression of neuron-specific transcription factors for neurogenesis (Qian et al., 2020). Furthermore, striatal astrocyte-to- dopaminergic neuron conversion also can be achieved by overexpression the neuron-specific transcriptional genes such as Ascl1, NeuroD1, Lmx1a, and miR-218 (Fyfe, 2017; Rivetti di Val Cervo et al., 2017). Str, Striatum; SN, Substantia Nigra; PTB, Polypyrimidine Tract-Binding protein; TFs, Transcriptional Factors; ASOs, PTB antisense oligonucleotides.

Two recent studies now provide a very simple strategy to efficiently reprogram astrocytes into iDA neurons *in vivo*, which only need to deplete an RNA-binding protein called PTB (polypyrimidine tract-binding protein) encoded by a single gene, polypyrimidine tract-binding protein 1 (*Ptbp1*) in astrocytes (Arenas, 2020; Qian et al., 2020; Zhou et al., 2020; Jiang et al., 2021). Previous studies have shown that *Ptbp1* (PTB) expression is observed in most cell types, and the other two PTB family members in mammalian genomes are *Ptbp2* (also called nPTB or brPTB) which is exclusively expressed in the nervous system, and *Ptbp3* (also called as ROD1) which is mainly detected in immune cells (Hu et al., 2018). It has been certified that PTB-regulated loop is very important for neuronal induction, and nPTB-regulated loop for neuronal maturation (Hu et al., 2018; Qian et al., 2020). In non-neuronal cells, such as fibroblasts, astrocytes, PTB-regulated loop is closely related to neuronal induction. In this loop, RE1-silencing transcriptional factor (REST) complex prevents the expression of multiple neuron-specific transcriptional genes (for example Ascl1, NeuroD1) as well as miR-124 in non-neuronal cells. Although REST is also the target of miR-124 in such loop, such targeting is potently inhibited by PTB via direct competition of miR-124 targeting on REST components (Xue et al., 2013, 2016; Figure 1). Thus, knockdown of *Ptbp1* can promote miR-124 to efficiently target REST. Considering that miR-124 also is able to target PTB, the derepression of miR-124 can further inhibits PTB and REST, leading to the expression of neuron-specific transcription factors for neurogenesis. The PTB-miR-124-REST loop, which is self-sustainable and conserved in mammals, can be triggered by *Ptbp1* knockdown (Xue et al., 2016). Cultured mouse embryonic fibroblasts can be converted into functional neurons by downregulation of the expression of *Ptbp1* (Xue et al., 2013), however, human adult fibroblasts are only converted into immature neurons according to the same protocol (Xue et al., 2016). The mechanism is that in adult human fibroblasts, downregulation of PTB increases the expression of nPTB, and meanwhile, high expression nPTB suppresses the transcription activator BRN2 (encoded by *Pou3f2*) and miR-9, both of which are required for neuronal maturation (Xue et al., 2013, 2016). Therefore, adult human fibroblast-to- neuron conversion requires both PTB-miR124-REST loop for neuronal induction and nPTB-BRN2-miR-9 loop for neuronal maturation *in vitro* (Xue et al., 2016). In astrocytes, during neurogenesis from neural stem cells, nPTB induced by PTB knockdown may be immediately counteracted by miR-9 (Qian et al., 2020). Further investigations found that the PTB-regulated loop in astrocytes is similar to the one in fibroblasts and the nPTB-regulated loop in astrocytes is similar to the one in neurons, and transient high expression of nPTB is observed in PTB-deficient astrocytes. These results suggest that astrocytes can be converted into neurons by *Ptbp1* knockdown (Qian et al., 2020). Indeed, both isolated mouse and human astrocytes are successfully converted into mature neurons by transfection with a lentivirus vector expressing shRNA against *Ptbp1* (shPTB) *in vitro* (Qian et al., 2020). The same effect is achieved in astrocytes isolated from the mouse cortex by using the genome-editing technique CRISPR-CasRx to deplete *Ptbp1* mRNA (Zhou et al., 2020).

These induced dopaminergic neurons from reprogramming of astrocytes in the substantia nigra or striatum can improve motor function in a mouse model of PD (Arenas, 2020; Qian et al., 2020; Zhou et al., 2020; Jiang et al., 2021). Qian et al. transduced mouse astrocytes in the substantia nigra with an adeno-associated virus (AAV; serotype2) vector to express a small hairpin RNA (shRNA) silencing *Ptbp1* expression (shPTB) (Qian et al., 2020). Qian et al. (2020) also synthesized PTB antisense oligonucleotides (ASOs) which is essential short nucleic acids and can bind to *Ptbp1* mRNA, thus preventing its translation into PTB protein (Qian et al., 2020). Notably, the local transient injection of ASOs in the substantia nigra also could generate dopaminergic neuron like cells and promoted the recovery of motor functions in PD mice (Qian et al., 2020). By contrast, Zhou et al. (2020) designed an AAV expressing CasRx and two guide RNAs (gRNAs) targeting *Ptbp1* mRNA to transfect astrocytes in the striatum. The results of Qian et al. (2020) suggested that the brain-region-specific transcription factors play crucial roles in the astrocyte-to-dopaminergic neuron conversion. However, because the region-specific transcription factors in the striatum are different from in the substantia nigra, this mechanism is not suitable to explain that striatal astrocytes can be converted into dopaminergic neurons in Zhou and colleagues’ study (Arenas, 2020; Qian et al., 2020; Zhou et al., 2020). Additionally, another important promoter on progress of astrocyte-to-dopaminergic neuron conversion is the local brain environment including various local brain-derived factors. Qian et al. (2020) results showed that the efficiency of astrocyte-to-dopaminergic neuron conversion was higher *in vivo* than *in vitro*. Beyond that, Zhou et al. (2020) found that Müller glia also could be converted into functional retinal ganglion cells in a mouse model of NMDA-induced retinal injury by AAV-GFAP-CasRx-*Ptbp1*, leading to alleviate symptoms caused by the loss of retinal ganglion cells. Besides, *in vitro* human fetal cortical astrocytes could be converted into different neurons by depleting *Ptbp1*, such as glutamatergic neurons (Qian et al., 2020). The above results indicate that glia-to-neuron conversion by PTB deleption may have a therapeutic potential for PD or other neurodegenerative diseases, even neuronal injuries such as trauma, tumor, or stroke.

However, many questions of *in situ* direct conversion of astrocytes to functional dopaminergic neurons remain to be answered (Arenas, 2020). For instance, astrocytes with depletion of PTB are also converted to other neuron types, such as glutamatergic neurons, GABAergic neurons and interneurons, in addition to dopaminergic neurons (Qian et al., 2020; Zhou et al., 2020). Given that *Ptbp1* is also expressed in other midbrain cell types (La Manno et al., 2016; Arenas, 2020), such as endothelial and pericyte cells, ependymal cells and microglia, therefore, there is an intriguing question whether these cells can also been converted into dopaminergic neurons by PTB depletion in animal models of PD. Moreover, although more than half of the fibers project to the striatum were from induced dopaminergic neurons, the vast majority of converted dopaminergic neurons project to the septum, rather than the striatum (Arenas, 2020; Qian et al., 2020). Besides, ectopic location or project of induced dopaminergic neurons might cause side effects, such as dyskinesia because of random and irregular release of dopamine. Although the simplicity of this gene-therapy approach to neuron replacement *in vivo* makes it very attractive, many questions remain to be answered, and it must be cautiously optimistic for the development of regenerative medicine for neurological disorders such as PD.

## *In vivo* Astrocyte-to-Neuron Conversion for AD

Alzheimer’s disease (AD), is the most prevalent neurodegenerative disease accounting for 60–80% of dementia cases, and it is manifested by memory loss and a decline in cognitive functions (Alzheimer’s disease facts and figures, Alzheimers Dement, 2021). Generally, the typical pathological characteristics of AD occur many years before the onset of clinical symptoms with the progressive accumulation of extracellular amyloid β (Aβ) plaques outside neurons and hyperphosphorylated tau (p-tau)-composed neurofibrillary tangles (NFTs) inside neurons, accompanied by neuronal death, synapses loss and global brain atrophy (Spires-Jones and Hyman, 2014; Zhang T. et al., 2021). It has been shown that there are several mechanisms involved in AD, such as inflammation and immune activation, lipid metabolism, endosomal vesicle recycling, and autophagy (Zhang T. et al., 2021). So far, no effective treatment is available to stop or even slow the progression of AD. Because AD is a disorder with extensive neurodegeneration, it is very difficult for cell transplantation to every area of the degenerating brain.

As for *in vivo* astrocyte reprogramming, it has been reported that a retrovirus expressing NeuroD1 under the control of human GFAP promoter, was constructed, and overexpression of NeuroD1 was capable of reprogramming reactive astrocytes into functional neurons in the adult mouse cortex in a mouse model of AD (Guo et al., 2014). NeuroD1-converted neurons had the ability of spontaneous and evoked synaptic responses by electrophysiological recordings. Interestingly, astrocytes were mainly converted into glutamatergic neurons whereas NG2 cells were reprogrammed into GABAergic neurons except glutamatergic neurons following NeuroD1 expression, suggesting that different glial cells may be involved in different neuronal fate due to lineage differentiation (Guo et al., 2014). Surprisingly, reactive astrocytes are much more competent and easily reprogrammed into neurons than quiescent astrocytes (Guo et al., 2014). Moreover, when cultured human astrocytes were infected with NeuroD1-retrovirus, they also can be efficiently reprogrammed into neurons, and the majority of NeuroD1-converted neurons were glutamatergic neurons (Guo et al., 2014). Unfortunately, the *in vivo* reprogramming of glia cells to neurons could not ultimately rescue behavioral deficits in a mouse model of AD, such as cognitive impairment.

Generally, the master transcription factors including NeuroD1, Sox2, Ngn2, and Ascl1 are closely related to guide the glia converted neuronal fate (Berninger and Jessberger, 2016; Li and Chen, 2016). NeuroD1 as a bHLH proneural transcription factor, is essential for embryonic brain development and adult neurogenesis (Cho and Tsai, 2004; Gao et al., 2009; Kuwabara et al., 2009), and can induce terminal neuronal differentiation (Boutin et al., 2010). The potentially mechanism is associated with the activation of downstream neural transcription factors (Gao et al., 2009; Kuwabara et al., 2009; Boutin et al., 2010), regulation of the Sonic hedgehog (SHH) signaling pathway (Sirko et al., 2013; Yang et al., 2019), and epigenetic modulation (Matsuda et al., 2019).

## *In vivo* Astrocyte-to-Neuron Conversion for HD

Huntington’s disease (HD) is an autosomal dominant disease characterized by the degeneration of GABAergic medium spiny neurons (MSNs) in the striatum and other brain regions, leading to progressive motor, cognitive, and psychiatric symptoms (Wu et al., 2020). It has been shown that 95% of the total neurons within the human striatum are MSNs (Reinius et al., 2015), and MSNs are susceptible to mutant huntingtin protein (mHtt) (Rikani et al., 2014). The accumulation and aggregation of mHtt in MSNs was discovered during early degeneration in the striatum of the patients with HD (Rikani et al., 2014). Previous studies have shown that both cell transplantation (Ma et al., 2012) and gene therapy (Yang S. et al., 2017) that can reduce the mHtt level, can promote neurological function recovery in animal models of HD.

To date, MSNs have been reprogrammed from human fibroblast cells *in vitro* (Victor et al., 2014). It has been reported that Dlx2 is important for generating GABAergic neurons (McKinsey et al., 2013; Yang N. et al., 2017). A lentiviral vector with high expression of miR-9/9^∗^-124 plus transcription factors Dlx1 and Dlx2 under a doxycycline (Dox)-inducible promoter was generated, and human fibroblasts were converted to striatal neurons after infection with these lentiviral vector (Victor et al., 2014). Recent report has shown that the AAV (serotype 2/5, rAAV2/5) with Cre-FLEx system was constructed, and it contained a vector expressing Cre recombinase under the control of GFAP promoter and FLEx vectors possessing an inverted coding sequence of NeuroD1-P2A-mCherry or Dlx2-P2A-mCherry. Notably, *in vivo* direct conversion of striatal astrocytes into MSNs has been achieved by this AAV-mediated ectopic expression of NeuroD1 and Dlx2 transcription factors (Wu et al., 2020). Those converted MSNs are electrophysiologically functional and forming synaptic circuits with other neurons, also can project their axon nerve terminals to substantia nigra pars reticulata and the external globus pallidus (Wu et al., 2020). Moreover, *in vivo* regeneration of MSNs in the striatum could reduce the striatum atrophy, and improve the motor functions in the R6/2 HD mouse model. In addition, the life span of the R6/2 HD mouse was significant extension after NeuroD1 + Dlx2 gene therapy treatment (Wu et al., 2020).

## *In vivo* Astrocyte-to-Neuron Conversion for ALS

Amyotrophic lateral sclerosis (ALS), also called Lou Gehrig disease, is a motor neuron degenerative disease, affecting mainly upper and lower motor neurons (MNs) in the motor cortex, brain stem, and spinal cord (Ferraiuolo et al., 2011; Meyer et al., 2014). Transactivation response DNA-binding protein 43 kDa (TDP-43)-positive cytoplasmic inclusions and bunina bodies are often presented in the lost motor neurons of patients with ALS (Neumann et al., 2006; Barp et al., 2020). The typical clinical symptoms are muscular atrophy and paralysis, ultimately leading to death caused by respiratory failure (Ferraiuolo et al., 2011; Meyer et al., 2014; Brown and Al-Chalabi, 2017). Indeed, ALS is caused by multiple factors, such as older age, household heredity factors, environmental and lifestyle factors (Brown and Al-Chalabi, 2017), and so far, ALS has been considered as a multi-system disorder, not just a motor neuron disease, and the pathogenesis of ALS still remains unclear. It has been demonstrated that many factors, such as neuroinflammation, glutamate-induced excitotoxicity, mitochondrial dysfunction, and so on, can damage the neuro-muscular junction integrity and retrograde axonal degeneration, ultimately resulting in motor neuronal degeneration (Brown and Al-Chalabi, 2017; Liu and Wang, 2017). The technology application of astrocyte-to-neuron-conversion may bring hope to develop new therapeutic strategies for ALS.

Recently, Zhao et al. (2020) reported that both human astrocytes and mouse astrocytes from an ALS mouse model carrying a SOD1 mutation could be rapidly and efficiently converted into motor neuron-like cells by treatment with defined small molecules (Kenpaullone, Forskolin, Purmorphamine, Retinoic acid) *in vitro*. These induced motor neuron-like cells expressed motor neuron markers and had the electrophysiological properties of neurons (Zhao et al., 2020).

## *In vivo* Astrocyte-to-Neuron Conversion for Other CNS Diseases

Given astrocytes are widely distributed in the CNS, *in vivo* astrocyte-to-neuron conversion is associated with other CNS disease. For example, the single neural transcription factor NeuroD1-based gene therapy can successfully reprogram astrocytes into functional neurons in damaged spinal cord (Puls et al., 2020). More recently, AAV-based NeuroD1 gene therapy can regenerate a large number of functional neurons, reduce microglia and macrophage activation, and protect parvalbumin interneurons in the converted areas, leading to restoring brain functions after ischemic injury in adult mice and non-human primates (Chen et al., 2020; Ge et al., 2020; Zhang X. et al., 2021). Many groups have also successfully converted astrocytes into proliferative neuroblasts by overexpression of transcription factor Sox2, and then further can be differentiated into neurons in mouse brain and injured adult spinal cord (Niu et al., 2013, 2015; Su et al., 2014; Islam et al., 2015; Wang et al., 2016).

## Advantages and Challenges of *In vivo* Astrocyte-to-Neuron Conversion

Recently, *in vivo* astrocyte-to-neuron conversion technology provides an alternative approach to regenerate functional new neurons in adult mammalian brains by directly reprogramming local astrocytes into neurons (Fyfe, 2017; Rivetti di Val Cervo et al., 2017; Qian et al., 2020; Zhou et al., 2020). This *in situ* neuron conversion technology not only provides an alternative strategy for regenerating functional new neuron by directly converting local astrocytes into neurons, but also avoid the disadvantages from the transplantation process in CNS. Given that astrocytes are abundant in CNS, the reprogramming of astrocytes into functional neurons *in situ* provides a new potential strategy to restore lost neuronal function, and this approach has no immunosuppression, no ethical concerns, as well as no cell transplantation. Beyond that, *in vivo* astrocyte-to-neuron conversion provide the possibility of targeting larger astrocyte numbers, perhaps it is possible that replace the widespread loss of neurons in neurodegenerative diseases, such as AD, which transplantation can hardly do.

Although *in vivo* astrocyte-to-neuron conversion technology have a promising future in the development of regenerative medicine for neurodegenerative diseases, there are some challenges and limitations. Indeed, these approaches are still in their infancy (Gascón et al., 2017). It is unclear whether human astrocytes can also be converted into neurons by these approaches *in vivo*, if it success, whether the converted neurons are the wanted neuron type, can survive over long periods and integrate to neighbor neurons. As for the neurodegeneration caused by gene mutations, such as familial neurodegenerative diseases (Meyer et al., 2014; Przedborski, 2017; Singleton and Gasser, 2020; Wu et al., 2020; Zhang T. et al., 2021), the gene mutation is still present under certain genetic background, and these converted neurons from astrocytes might eventually degenerate. The efficiency of the existing direct lineage reprogramming strategy *in situ* still needs to be improved to meet clinical requirements. There are many varieties of influencing factors on reprogramming astrocytes into neurons *in vivo*. First, pathological environments, such as in AD or PD, are filled with various deleterious factors, such as ROS, inflammatory cytokines (Xu et al., 2016; Singh et al., 2019; Kwon and Koh, 2020) that all affect the neuronal reprogramming, survival and integration *in vivo*. Second, given that reprogramming astrocytes into neurons *in vivo* has to take place in a complex mixture of many various cell types, and the influences of the neighboring cells play an important role in the neuronal conversion and correct integration of the converted neurons (Gascón et al., 2017). Third, considering that neurodegenerative disorders tend to occur in older people, the age-related limitations of reprogramming must be taken into consideration, for example, astrocyte senescence is observed in patients of AD (Bhat et al., 2012), and PD (Chinta et al., 2018). Fourth, the mechanisms underpinning fate conversion and neuronal subtype identity is still unclear. Moreover, it remains to be a question whether it is possible to use small molecule strategy, rather than gene therapy, to direct the neuronal reprogramming *in vivo*. Thus, much more work remains to be done to develop a non-invasive manner approach for fully using the power of neuronal reprogramming in neuronal replacement therapies. Last but not least, it is exciting for *in vivo* astrocyte-to-neuron conversion has been successful in rodent models of PD (Rivetti di Val Cervo et al., 2017; Qian et al., 2020; Zhou et al., 2020), AD (Guo et al., 2014), and HD (Wu et al., 2020), even in non-human primates with ischemic stroke (Ge et al., 2020; Zhang X. et al., 2021), and it will be looking forward to the clinical application of direct neuronal reprogramming in neurodegenerative diseases.

## Author Contributions

YW wrote the first draft. XZ and FC designed the figure. NS and JX reviewed and critiqued the manuscript. All authors contributed to the article and approved the submitted version.

## Conflict of Interest

The authors declare that the research was conducted in the absence of any commercial or financial relationships that could be construed as a potential conflict of interest.

## Publisher’s Note

All claims expressed in this article are solely those of the authors and do not necessarily represent those of their affiliated organizations, or those of the publisher, the editors and the reviewers. Any product that may be evaluated in this article, or claim that may be made by its manufacturer, is not guaranteed or endorsed by the publisher.
